# An open database of resting-state fMRI in awake rats

**DOI:** 10.1016/j.neuroimage.2020.117094

**Published:** 2020-06-28

**Authors:** Yikang Liu, Pablo D. Perez, Zilu Ma, Zhiwei Ma, David Dopfel, Samuel Cramer, Wenyu Tu, Nanyin Zhang

**Affiliations:** a Department of Biomedical Engineering, The Pennsylvania State University, University Park, PA, 16802, USA; b Neuroscience Program, The Huck Institutes of the Life Sciences, The Pennsylvania State University, University Park, PA, 16802, USA

**Keywords:** Database, Resting-state fMRI, Awake, Rat

## Abstract

Rodent models are essential to translational research in health and disease. Investigation in rodent brain function and organization at the systems level using resting-state functional magnetic resonance imaging (rsfMRI) has become increasingly popular. Due to this rapid progress, publicly shared rodent rsfMRI databases can be of particular interest and importance to the scientific community, as inspired by human neuroscience and psychiatric research that are substantially facilitated by open human neuroimaging datasets. However, such databases in rats are still rare. In this paper, we share an open rsfMRI database acquired in 90 rats with a well-established awake imaging paradigm that avoids anesthesia interference. Both raw and preprocessed data are made publicly available. Procedures in data preprocessing to remove artefacts induced by the scanner, head motion and non-neural physiological noise are described in details. We also showcase inter-regional functional connectivity and functional networks obtained from the database.

## 1. Introduction

Resting-state functional magnetic resonance imaging (rsfMRI) measures spontaneous brain activity in the absence of explicit external tasks or stimuli based on the blood-oxygenation-level dependent (BOLD) contrast (Biswal et al., 1995). Due to its high spatial resolution and whole-brain coverage, this method has tremendously advanced our understanding of human brain networks in terms of the function (Greicius et al., 2003; Hampson et al., 2002; Lowe et al., 1998), organization (Fox et al., 2005; Wang et al., 2010), development and aging (Dosenbach et al., 2010; Fair et al., 2008), as well as genetic basis (Fu et al., 2015; Wiggins et al., 2012), and has provided a potential biomarker that can be used to track the progress of brain disorders, and evaluate the efficacy of treatment (Lee et al., 2013).

Compared to human research, studying rodent models using rsfMRI has unique advantages. First, environmental and genetic background are relatively uniform, making it easier to separate their influences on brain networks and function (Gorges et al., 2017). Second, fMRI can be combined with cutting-edge neuroscience techniques such as electrophysiology (Majeed et al., 2011; Pan et al., 2010; Sloan et al., 2010), optogenetics (Desai et al., 2011; Lee et al., 2010; Liang et al., 2015b), calcium signal recording (Liang et al., 2017; Schlegel et al., 2018) and Designer Receptors Exclusively Activated by Designer Drugs (DREADDs) (Grayson et al., 2016), which can facilitate bridging wide-range information from the cellular to systems levels. Applying rsfMRI in transgenic rodent models can further link imaging discoveries to neural mechanisms at the genetic and molecular levels (Asaad and Lee, 2018). Third, rodent rsfMRI studies have high translational value. Using rsfMRI and other techniques, functional networks such as the thalamocortical and default mode networks (DMNs) have been identified in rodents that bear high anatomical resemblance as those in humans (Liang et al., 2013; Lu et al., 2012a,; Stafford et al., 2014). Topological organization such as small-worldness and rich-club organization is also well conserved in humans, non-human primates and rodents (Bullmore and Sporns, 2009; Liang et al., 2011; Ma et al., 2018a; van den Heuvel and Sporns, 2011). Taken together, rsfMRI provides a powerful tool in characterizing rodent models that complement human studies.

Despite these significant potentials, there is a large disparity in the number of publications between animal and human studies using rsfMRI. A major challenge is that anesthesia is often used in animal rsfMRI experiments to immobilize animals. It becomes increasingly clear that anesthesia changes physiological conditions (Tsukamoto et al., 2015), neurovascular coupling (Devor et al., 2007), brain metabolism (Hyder et al., 2002), and function of brain circuits and networks (Hamilton et al., 2017; Liang et al., 2011; Lu et al., 2007; Ma et al., 2017). In addition, the effects of anesthesia vary across different anesthetic agents and dosages (Grandjean et al., 2014; Hamilton et al., 2017), making it difficult to integrate data from different labs using different anesthesia protocols. Therefore, to avoid these issues it is important to image animals at the awake state when studying brain function and network.

Since late 1990s, several labs, including ours, have established an awake animal rsfMRI paradigm that allows the brain function to be studied without the interference of anesthesia (Zhang et al., 2010; Bergmann et al., 2016; Chang et al., 2016; Yoshida et al., 2016; Lahti et al., 1998; Ferris et al., 2006). In this paradigm, animals are acclimated to the MRI scanning environment to minimize their stress and motion during imaging (King et al., 2005). We have demonstrated that this acclimation procedure, unlike studies of chronic stress that use prolonged daily restraint, does not induce chronic stress, nor does it interact with other stressors (Dopfel et al., 2019; Liang et al., 2014). By utilizing this method, we have investigated spatiotemporal dynamics of individual neural circuits (Liang et al., 2013, 2012a) and whole-brain networks (Liang et al., 2011; Liu and Zhang, 2019; Ma and Zhang, 2018; Ma et al., 2018b, 2020). This method has also be employed to reveal changes in whole-brain connectivity architecture during brain development (Ma et al., 2018a), under anesthesia (Hamilton et al., 2017; Liang et al., 2015a, 2012b; Ma et al., 2017; Smith et al., 2017), as well as neuroplastic changes induced by traumatic stress (Dopfel et al., 2019; Liang et al., 2014) and drugs (Crenshaw et al., 2015; Pérez et al., 2018; Roses et al., 2014). Moreover, the same approach has been applied to other species including mice, rabbits, monkeys and dogs (Berns et al., 2012; Goense and Logothetis, 2008; Schroeder et al., 2016; Desai et al., 2011; Yoshida et al., 2016). Taken together, these studies have demonstrated the validity and value of the awake fMRI approach.

Inspired by human neuroscience and psychiatric research that are substantially facilitated by open human neuroimaging datasets, here we share an open database, which contains 175 rsfMRI scans from 90 rats acquired in the awake state, to the public. We provide both raw and preprocessed data. Some results obtained from routine analyses are demonstrated.

## 2. Methods and materials

### 2.1. Animals

Data were acquired in 90 adult male Long-Evans rats (300 g–500 g), part of which were used in previous publications (Dopfel et al., 2019; Liu and Zhang, 2019; Ma et al., 2018a; Ma and Zhang, 2018; Ma et al., 2018b). All rats were housed in Plexiglas cages (two per cage) with food and water provided *ad libitum*. A 12 h light: 12 h dark schedule and ambient temperature between 22 °C and 24 °C were maintained. All experiments were approved by the Institutional Animal Care and Use Committee (IACUC) of the Pennsylvania State University.

### 2.2. Acclimation procedure

The purpose of this procedure is to acclimate the animal to the restraining system as well as the noisy and confined environment inside the MRI scanner. Details of the acclimation procedure can be found in our previous publications (Dopfel and Zhang, 2018; Gao et al., 2017). Briefly, EMLA cream (2.5% lidocaine and 2.5% prilocaine) was applied topically to the nose, jaw, and ear areas to relieve any discomfort associated with the restrainer 20 min prior to the procedure. The animal was then briefly anesthetized with 2–4% isoflurane and placed in a head restrainer, in which the teeth and nose were secured by a bite bar and a nose bar, respectively, and ears were secured by adjustable ear pads. Forepaws and hindpaws were loosely taped to prevent the animal from accidental self-injury. After that, the body was placed in a Plexiglas body holder with the shoulders secured by a pair of shoulder bars. The whole system allowed unrestricted breathing. Isoflurane was discontinued after the setup. The restrainer was then fixed to a body holder. After the animal woke up, the system was placed into a black opaque chamber where the prerecorded sound from various imaging sequences was played. The animal was acclimated for 7 days with an incremental exposure time up to 60 min (i.e. 15, 30, 45, 60, 60, 60 and 60 min from Day 1 to Day 7, respectively). This procedure has also been employed by several other groups for awake rodent fMRI (Bergmann et al., 2016; Chang et al., 2016; Yoshida et al., 2016).

### 2.3. Data acquisition

Data were acquired on a 7T Bruker 70/30 BioSpec running ParaVision 6.0.1 (Bruker, Billerica, MA) at *the High Field MRI Facility* at the Pennsylvania State University. Similar to the setup in the acclimation procedure, the animal was briefly anesthetized with 2–4% isoflurane and were placed in a head restrainer integrated with a birdcage head coil. The isoflurane was discontinued once the setup was finished. rsfMRI acquisition started when the animal was fully conscious (usually within 10–15 min). A single-shot gradient-echo echo-planar imaging (GE-EPI) sequence was used with the following parameters: repetition time (TR) = 1000 ms; echo time (TE) = 15 ms; matrix size = 64 × 64; field of view (FOV) = 3.2 × 3.2 cm^2^; slice number = 20; slice thickness = 1 mm; slice gap = 0 mm; flip angle = 60°, 600, 900, or 1200 volumes per scan, 2 to 4 scans per animal. A representative raw EPI image is shown in Fig. S1a. Anatomical images were also acquired with a rapid imaging with refocused echoes (RARE) sequence with the following parameters: TR = 1500 ms; TE = 8 ms; matrix size = 256 × 256; FOV = 3.2 × 3.2 cm^2^; slice number = 20; slice thickness = 1 mm; slice gap = 0 mm.

### 2.4. Data preprocessing

**Fig. 1.**
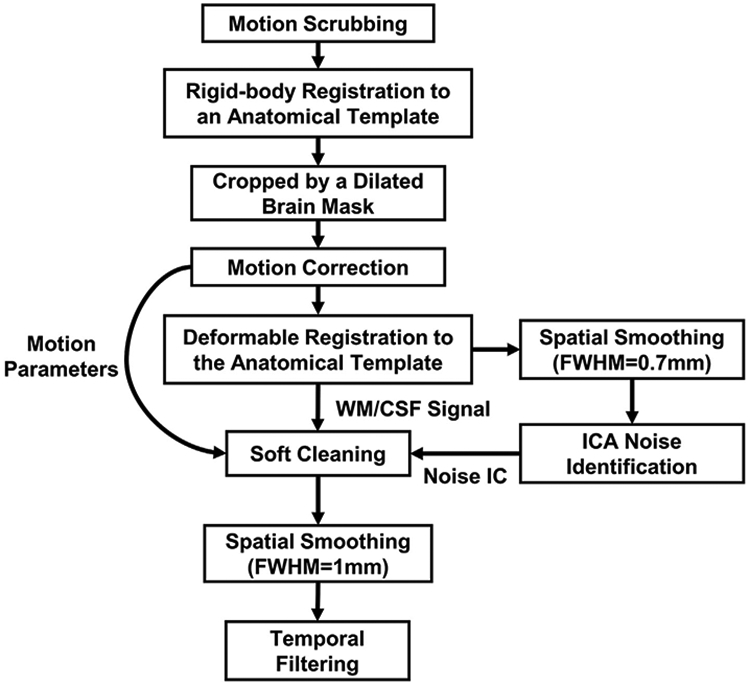
rsfMRI data preprocessing pipeline.

The preprocessing procedures generally followed those commonly used in human rsfMRI data, but were adapted to optimize the performance for rat rsfMRI data. The preprocessing pipeline is outlined in Fig. 1, which included 9 steps:

1. Volumes with excessive motion were discarded (i.e. scrubbing).
2. rsfMRI images were manually co-registered to an anatomical template with rigid-body transformation.
3. Co-registered images were cropped by a dilated brain mask to facilitate motion correction.
4. Co-registered images were corrected for head motion and motion parameters were recorded.
5. Motion-corrected images were normalized to the anatomical template with deformable registration.
6. Non-neural artefacts were identified with independent component analysis (ICA) on smoothed normalized images (FWHM = 0.7 mm). Time courses of noise independent components (ICs) were recorded.
7. Unsmoothed normalized images were softly cleaned by regressing out noise IC time courses, motion parameters and the nuisance signals from the white matter (WM) and cerebral spinal fluid (CSF).
8. Softly cleaned images were spatially smoothed (FWHM = 1 mm).
9. Spatially smoothed images were temporally bandpass filtered.

All source codes used for preprocessing can be downloaded from the GitHub repository: https://github.com/liu-yikang/rat_rsfmri_preprocessing. Details of each step were described below.

### 2.5. Motion scrubbing and co-registration

First, motion was evaluated by calculating the relative framewise displacement (FD) of each rsfMRI volume (Power et al., 2012). Specifically, the geometric transformation from each frame (i.e. 3D volume) to the first frame was evaluated by the built-in function *imregtform* in MATLAB (The Mathworks Inc., Natick, MA, USA) with six degrees of transformation considered (i.e. rigid-body transformation), including translations in the three orthogonal axes (translation distances for the frame *i* are denoted as *x*_*i*_, *y*_*i*_, and *z*_*i*_) and rotations around the three axes (rotation angles are denoted as *α*_*i*_, *β*_*i*_, and *γ*_*i*_). Then *FD*_*i*_ = ∣ *Δx_i_* ∣ + ∣ *Δy_i_* ∣ + ∣ *Δz_i_* ∣ + *r* · (∣ *Δα_i_* ∣ + ∣ *Δβ_i_* ∣ + ∣ *Δγ_i_* ∣), where *r* = 5 mm, which is approximately the distance measured from the cortex to the center of the rat head. Frames with *FD* > 0.2 mm and their neighbor frames were discarded. The first 10 frames of each scan were also discarded to ensure steady state of magnetization. Scans with less than 90% of the total number of frames left were excluded from further analysis. This procedure and parameters used can effectively minimize motion artefacts as confirmed in our previous studies (Liu and Zhang, 2019; Ma and Zhang, 2018). For the scans remained, 5.62 ± 2.39% (mean ± std) frames were scrubbed. The FD values of each scan were included in the corresponding .json file of the scan.

Next, the first frame of each rsfMRI scan was manually co-registered (i.e. aligned) to a T2-weighted anatomical template using an in-house software written in MATLAB. To ensure the quality of alignment, voxels at brain boundaries, ventricles, and WM in the anatomical template were displayed as landmarks on a graphical-user interface (Fig. S2). After the coregistration of the first frame, the same geometric transformation was applied to the remaining frames.

**Fig. 2.**
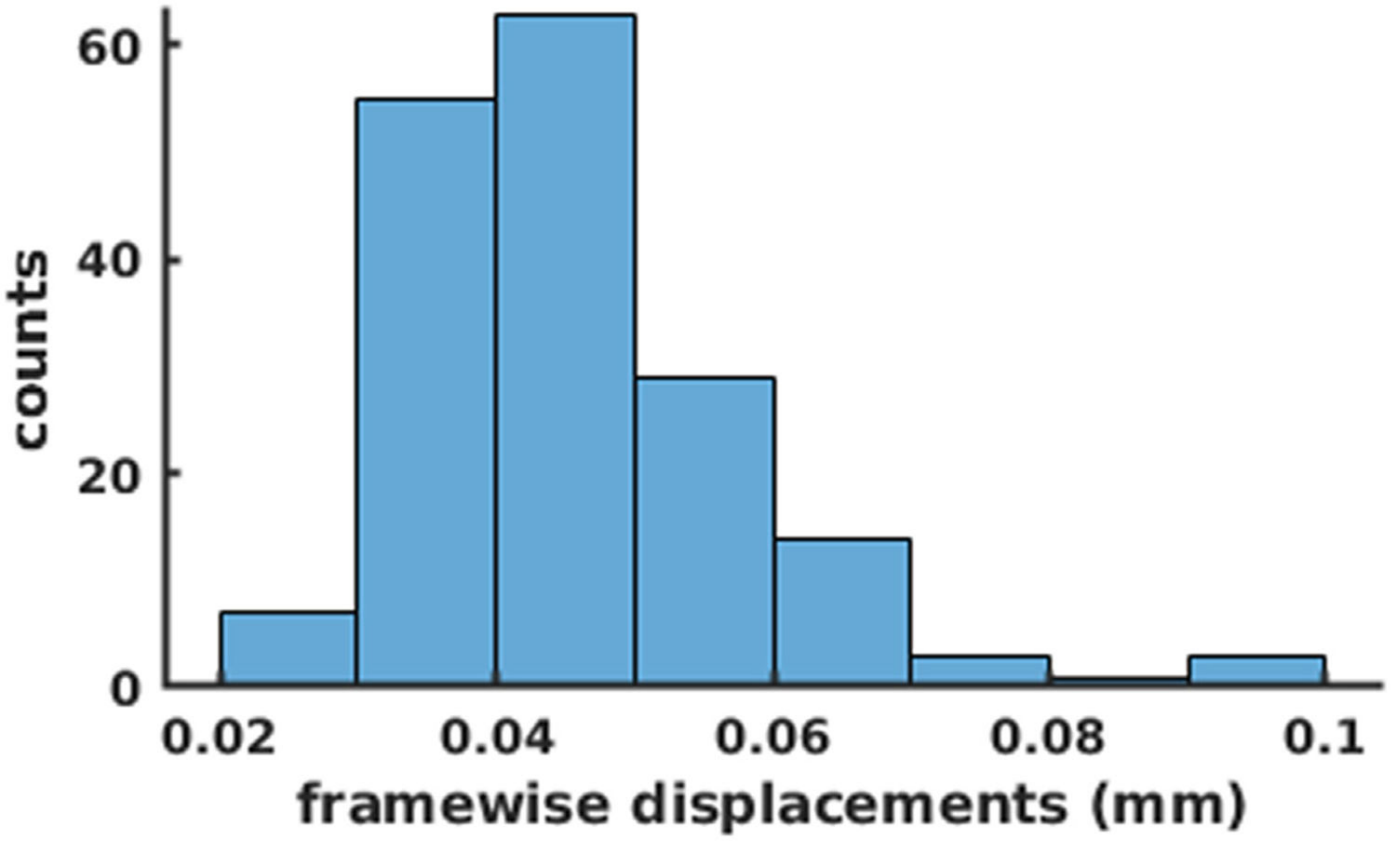
Distribution of averaged frame-wise displacement across scans.

Subsequently, head motions were corrected using SPM12 (http://www.fil.ion.ucl.ac.uk/spm/) with a dilated brain mask applied, in which each frame was co-registered to the first frame through a rigid-body transformation. Motion parameters were recorded for further use. The distribution of averaged FD across scans is displayed in Fig. 2 (mean: 0.0507 mm; median: 0.0416 mm).

Lastly, the motion-corrected images were registered to the anatomical template with SyN diffeomorphic transformation (*antsIntroduction.sh*) using ANTS (Advanced Normalization Tools, http://picsl.upenn.edu/software/ants/) (Avants et al., 2008). A representative example of deformable registration is shown in Fig. S1d.

### 2.6. ICA-based artefact identification

We used ICA to remove non-neural artefacts potentially related to motion, breathing, heartbeats, and/or scanner instability from spatially co-registered data. ICA-based artefact removal has been widely applied in human and rodent studies (Griffanti et al., 2015, 2014; Han et al., 2019; Salimi-Khorshidi et al., 2014; Smith et al., 2013). It leverages the independency between spatial and/or temporal patterns of the neural and non-neural components to separate them. In this method, users can manually identify each IC as real signal or noise based on their spatial, temporal, and spectral features. Standards of manually classifying ICs based on these features for the HCP data (Smith et al., 2013) were listed in Table 1 (Griffanti et al., 2017). Our specific procedure followed these guidelines, but was also adapted to the characteristics of rat data. We found one criterion not applicable to our rat data. In the HCP guideline, signal ICs tend to have a few large clusters, whereas noise ICs tend to have many small clusters. This criterion did not always hold true in rat data due to the relative portion of the cortex versus sub-cortex. The human brain is dominantly composed of the cortex, which contributes to most clustered structures in signal ICs. In contrast, 2/3 volume of the rat brain is sub-cortex that includes numerous heterogeneous nuclei. Thus, neural components in rats may not always display large clusters. Therefore, we grouped the HCP criteria into three categories: not applicable; applicable; confident, as listed in Table 1 and used the following criterion to label noise/signal ICs: an IC was classified as a noise component if it had one or two “confident” features or had at least two of the following three “applicable” features: 1) its spatial map is located predominately at white matters, ventricles, or brain boundaries; 2) its time course has sudden jumps; 3) the frequency spectrum is flat or dominated by very low or high frequency.

Prior to ICA, we spatially smoothed each frame with a Gaussian kernel (FWHM = 0.7 mm). The kernel size was empirically determined to improve the ICA performance, but still maintain the difference between neural and non-neural components. After that, spatial ICA was separately conducted on each scan using the GIFT ICA toolbox (Calhoun et al., 2009) with the number of ICs set at 50. Subsequently, we calculated the time courses of ICs by regressing their spatial maps against each frame.

**Fig. 3.**
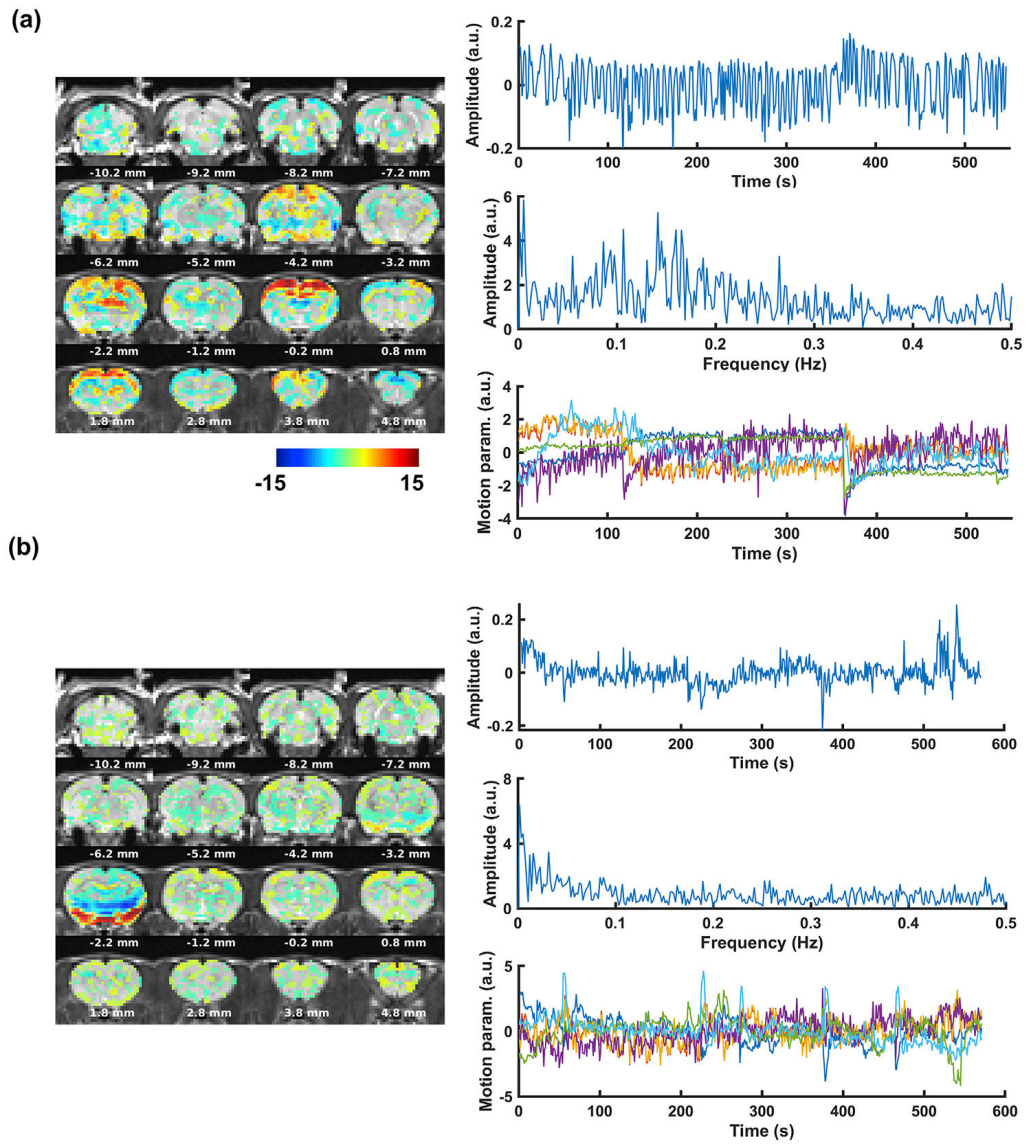
Two representative noise ICs from ICA-based cleaning.

We manually labeled ICs as signal or noise components for all scans of all animals based on the features of their spatial maps (z-scored, thresholded at z > 2), time courses, and spectra using the criteria mentioned above. 25.28 ± 9.32 (mean ± std) ICs were identified as noise components per scan. Two representative noise ICs are demonstrated in Fig. 3.

### 2.7. Soft cleaning, spatial smoothing, and temporal filtering

In the next step, we generated nuisance regressors from the signals in the WM and CSF regions using the CompCor method (Behzadi et al., 2007). The WM and CSF masks are shown in Fig. S3. We used the CSF mask in the SIGMA template, generated by thresholding the CSF probability map at 0.6, as it contains areas surrounding the pial surface (Barriere et al., 2019). The CompCor method used principal components (PCs), selected based on variance explained, of signals in WM and CSF voxels as regressors. First, 1000 datasets with the same data size were generated using Monte Carlo simulation (normally distributed). The *p* value of each PC in real data was determined by the portion of simulated datasets whose 1st PC had greater variance explained than the real-data PC. PCs with significant variance explained were selected (p < 0.05). In our data, 22.66 ± 6.19 (mean ± std) components were selected per scan.

Subsequently, we “softly” removed the noise ICs obtained using the method proposed by (Griffanti et al., 2014), and regressed out six motion parameters obtained in motion correction as well as the CompCor regressors from the rsfMRI data. In this method, only the unique parts of variance explained by the noise ICs were removed, whereas parts shared with the signal ICs were reserved. Briefly, first all ICA time courses and rsfMRI images were regressed by the corresponding motion parameters and CompCor regressors, resulting in regressed ICA time courses (*ICA*_*m*_) and regressed images (*Y*_*m*_). Second, we regressed *ICA*_*m*_ against *Y*_*m*_ to obtain the weight of unique contribution of each IC to the data: *β* = pinv(*ICA*_*m*_) · *Y*_*m*_. Third, we removed the unique contribution of the noise components from the data: *Y*_*clean*_ = *Y*_*m*_ - *ICA*_*m*_(noise) · *β*(noise).

Finally, all rsfMRI frames were spatially smoothed with a Gaussian kernel (FWHM = 1 mm), and the signal of each voxel in each rsfMRI scan was temporally filtered with a 4th-order bandpass Butterworth filter (0.01–0.1 Hz).

All raw and preprocessed data, the anatomical template, brain mask, and WM/CSF masks have been uploaded and can be freely downloaded (link: https://nitrc.org/projects/rat_rsfmri). The folder structure of raw and preprocessed data is described in the Appendix.

## 3. Results

### 3.1. Image quality

A representative raw EPI frame is shown in Fig. S1a. We calculated both spatial and temporal signal-to-noise ratio (sSNR and tSNR) for each scan. The sSNR was voxelwise determined using the 10th frame of the scan, calculated by the rsfMRI value of the voxel divided by the standard deviation of 1000 voxels outside of the brain, defined by two 5 × 5 voxel cubes at the left and right top corners in each slice. The tSNR was voxelwise calculated by the mean rsfMRI value divided by the standard deviation of the voxel’s time course. Both SNR maps were averaged across scans and displayed in Figs. S1b and S1c (left panels), respectively. In addition, sSNR and tSNR were averaged across all brain voxels, and the distributions of averaged within-brain SNRs across scans are shown in Figs. S1b and S1c (right panels), respectively.

### 3.2. Region-based correlational analysis

**Fig. 4.**
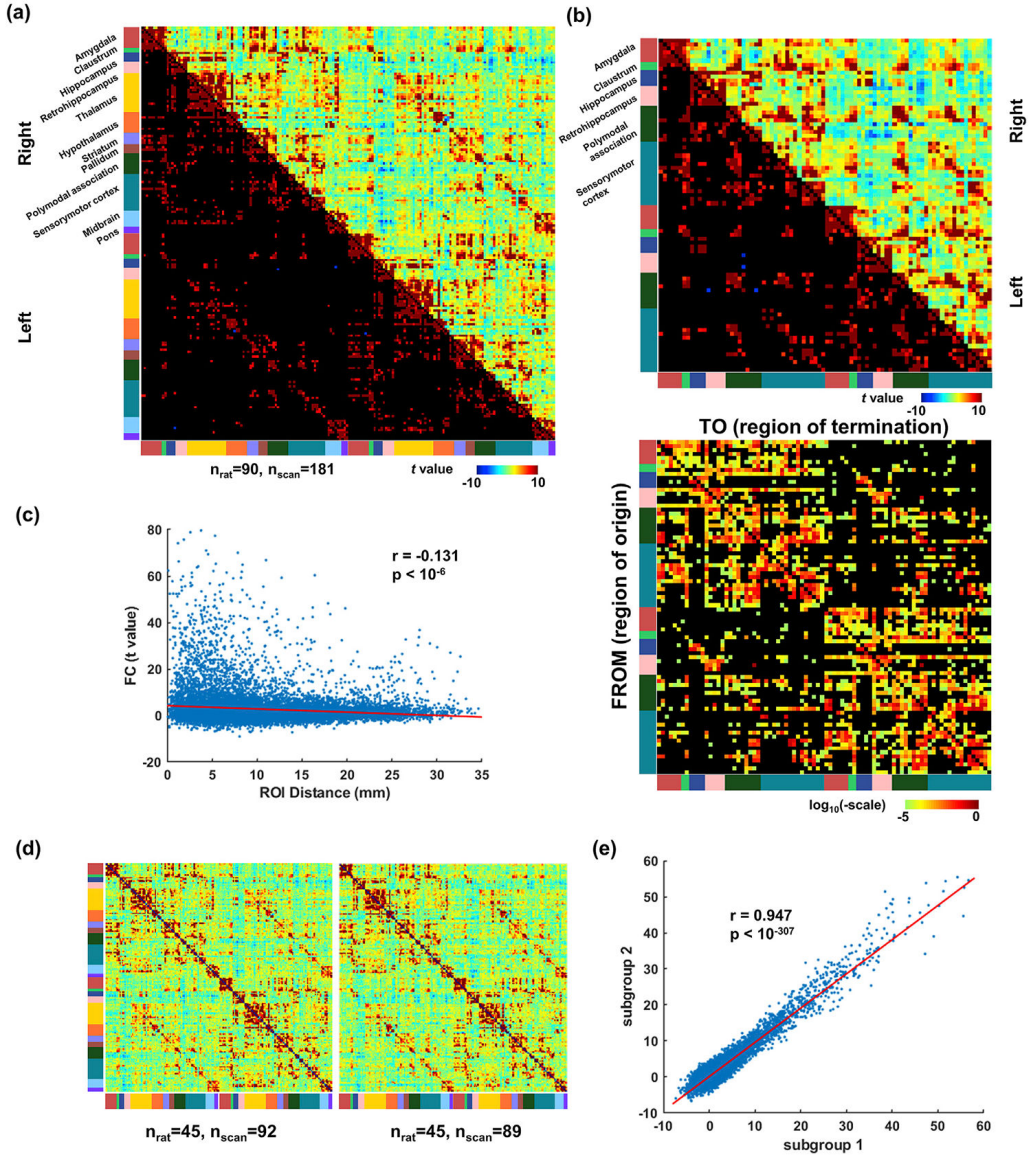
Pair-wise FC between ROIs. (a) FC matrix of all animals. The lower triangle shows entries (i.e. connections) with significant FC thresholded at FWER <0.05. (b) Cortical FC (upper panel) and cortical SC (lower panel) matrices. (c) The relationship between FC and ROI distance. (d) FC matrices of two randomly divided subgroups. (e) Correlation of the corresponding off-diagonal entries in the two matrices in (d) after regressing out ROI distance.

Fig. 4a shows the group-level pairwise FC between 180 unilateral regions of interest (ROIs) covering the whole brain. ROIs are defined based on Swanson atlas (Swanson, 2004), organized and color coded by the brain systems (i.e. color bars next to the FC matrix). The group-level FC (in *t* value) was calculated by fitting a linear mixed model (subject variability modeled as the random effect) to the FC of individual scans (i.e. one-sample *t* tests on the random effect), which was quantified by Fisher Z-transformed Pearson correlation coefficient of regionally averaged rsfMRI time courses between every two ROIs. To ensure the same degree of freedom of individual scans, scans with 600 or 900 vol were truncated into a 540-vol scan, and scans with 1200 vol were truncated into two 540-vol scans. This operation resulted in 181 scans for processing. The lower triangle shows entries (i.e. connections) with significant FC, thresholded by the familywise error rate (FWER) < 0.05 based on a permutation test, where ROI labels were independently shuffled for each scan in each permutation and the maximal *t* value of the resulting group-level ROI FC matrix was calculated. The permutation was repeated for 1000 times to form a null distribution of the maximal *t* value of each connection, and its FC was deemed significant when the real *t* value exceeded 95 percentile in the distribution. The density of significant FC was 10.35%.

We characterized our group-level FC matrix in several aspects. First, we compared cortical FC to cortical structural connectivity (SC) reported in (Swanson et al., 2017). ROIs in the SC matrix were merged to match ROIs in the FC matrix. Both matrices were shown in Fig. 4b, with the Jaccard index between supre-threshold cortical FC and cortical SC of 0.319 (p ≈ 0, permutation test). Fig. 4c shows a negative correlation (r = −0.139, p < 10^−6^) between FC and Euclidean ROI Distance, consistent with that reported in the mice brain connectome measured by rsfMRI (Grandjean et al., 2020). Fig. 4d shows the group-level reproducibility of FC, calculated by the similarity of FC matrices in two randomly divided subgroups. The correlation of the corresponding off-diagonal entries between the two matrices, after regressing out ROI distance, was 0.947 (p ≈ 0, Fig. 4e). The reproducibility of FC at the individual level was quantified by the correlation of off-diagonal entries between the FC matrix of each individual animal and that of the whole group (excluding the tested animal) after regressing out ROI distance. The averaged correlation value across animals was 0.430 ± 0.031 (mean ± std, p ≈ 0). Also to demonstrate the individual variability of FC, we show the distributions of FC between 12 ROIs across scans (Fig. S4), including unilateral anterior cingulate area (ACA), retrosplenial cortex (RSP), primary somatosensory cortex (SSp), dentate gyrus (DG), nucleus accumbens (ACB), and ventral anterio-lateral complex of thalamus (VAL).

**Fig. 5.**
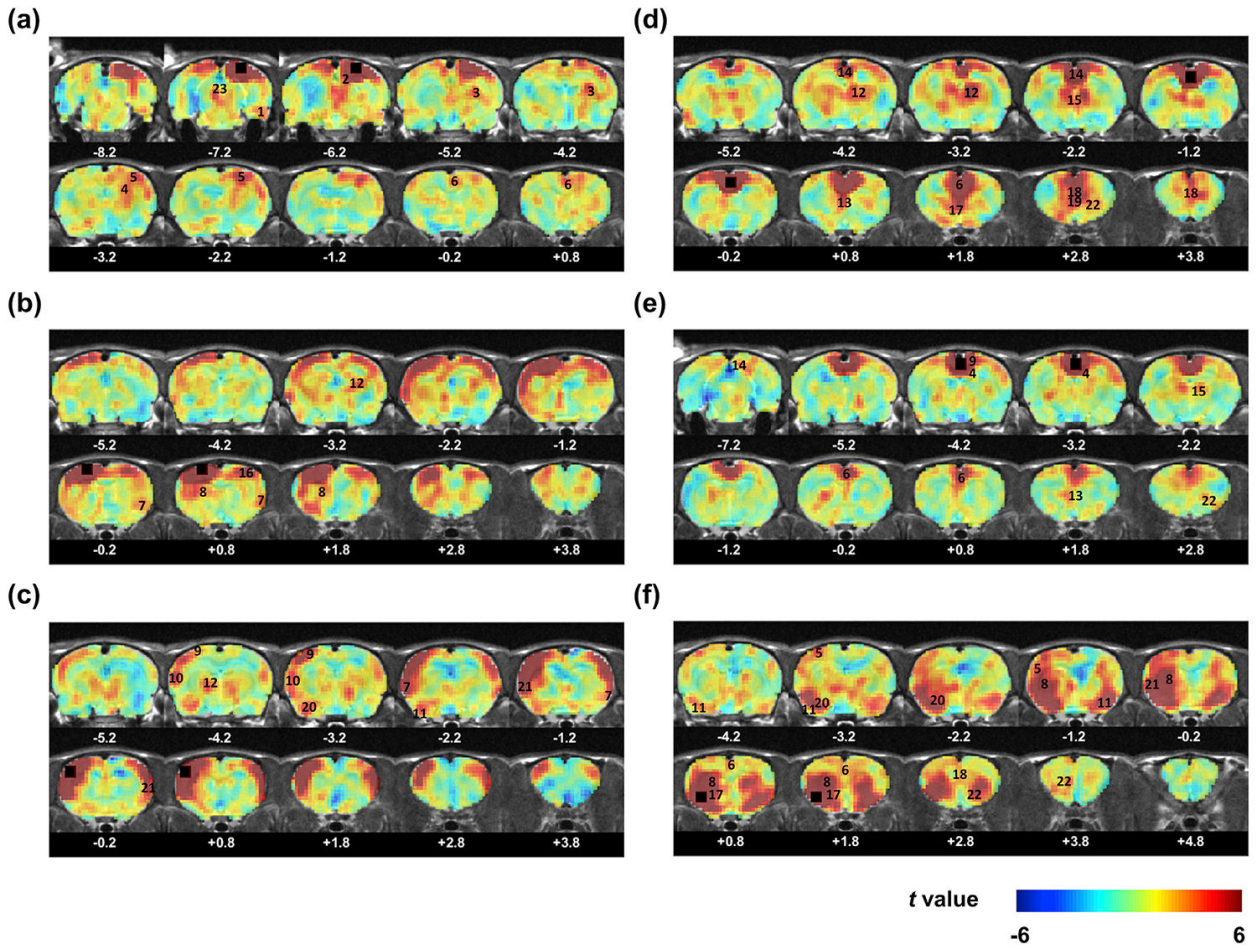
Representative seed maps. The seed regions are in (a) the visual cortex (VIS), (b) primary motor cortex (MOp), (c) primary somatosensory cortex (SSp), (d) anterior cingulate cortex (ACA), (e) retrosplenial cortex (RSP), and (f) insular cortex. The seed regions are marked with black boxes. 1. Entorhinal cortex (EnT); 2. Superior colliculus (SC); 3. Dorsolateral geniculate nucleus (DLG); 4. Dorsal hippocampus (dHC); 5. SSp; 6. ACA; 7. Insular cortex; 8. Dorsal striatum; 9. Parietal association area (PTA); 10. Auditory cortex (AUD); 11. Piriform cortex (PIR); 12. Dorsal thalamus; 13. Septum; 14. RSP; 15. Anterior thalamus; 16. MOp; 17. Ventral striatum; 18. Prelimbic cortex (PL); 19. Infralimbic cortex (IL); 20. Amygdala; 21. Secondary somatosensory cortex (SSs); 22. Orbital cortex (ORB); 23. Periaqueductal gray (PAG).

Fig. 5 shows a few examples of group-level seed maps, revealed by seed-based correlational analysis (hypothesis driven) with the seeds of 3 × 3 × 2 voxel cubes in the visual cortex (VIS), primary motor cortex (MOp), SSp, ACA, RSP, and insular cortex, respectively. Voxel-wise FC (in *t* value) was calculated in the same manner. All ROI seed maps can be downloaded from the database.

We further demonstrate the specificity of FC between selected seeds (Fig. S5), using the method described in (Grandjean et al., 2020). Here we used two groups of seed/ROI definitions. One group used ACA, RSP, SSp as the seed, specific ROI, and non-specific ROI, respectively, and the other group used SSp, contralateral SSp, and RSP as the seed, specific ROI, and non-specific ROI, respectively. These selections were based on the observed segregation between the default-mode network regions and the sensory cortex (Grandjean et al., 2020), as well as strong bilateral connectivity in the sensory cortex. For the ACA seed, 38.67%, 12.71%, 19.34%, and 29.28% of scans showed specific, unspecific, no, and spurious FC, respectively. For the SSp seed, 35.91%, 9.94%, 30.39%, and 23.76% of scans showed specific, unspecific, no, and spurious FC, respectively. These numbers are in line with the report in the literature (Grandjean et al., 2020).

### 3.3. ICA analysis

**Fig. 6.**
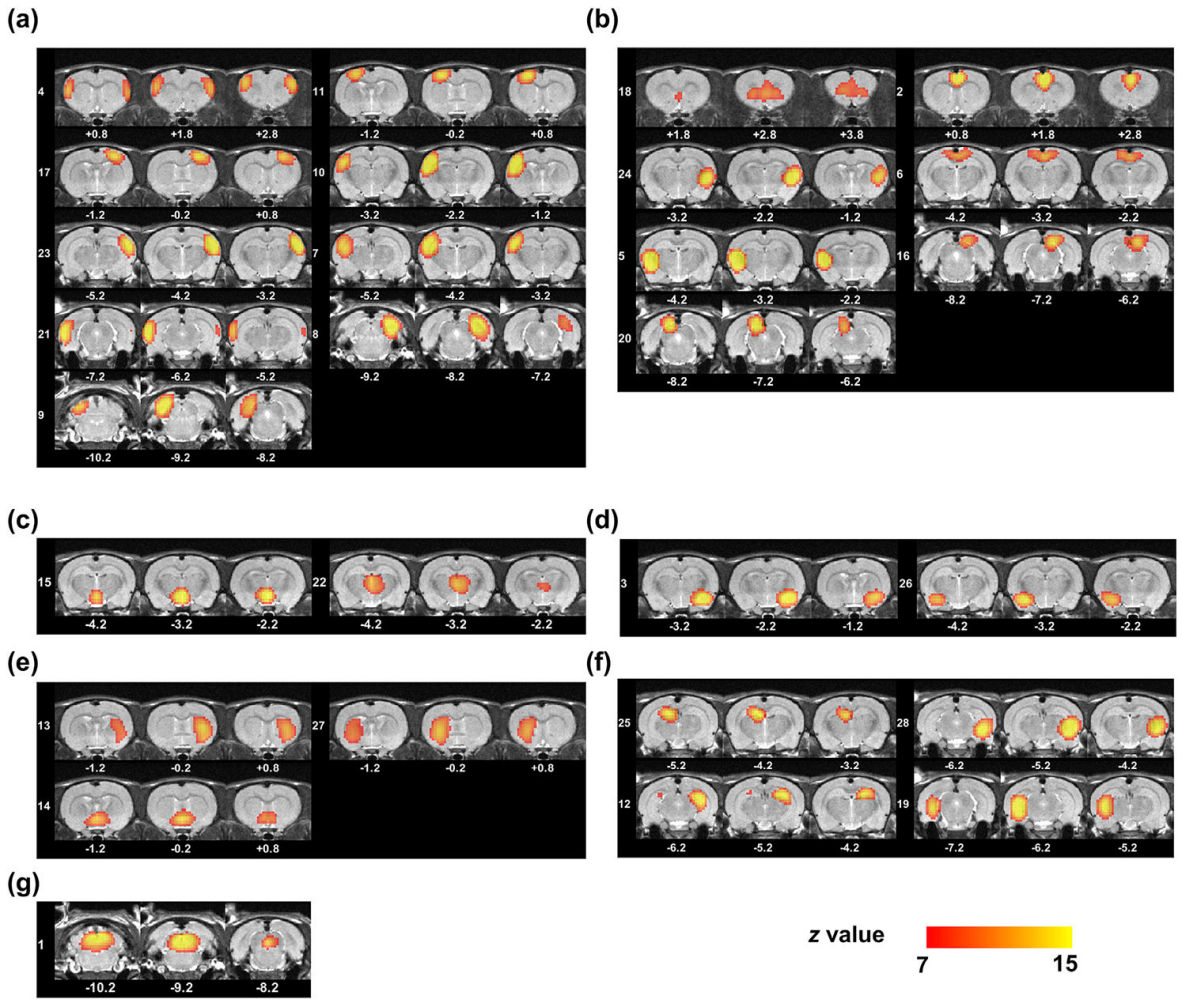
Spatial maps of 28 ICs generated by spatial group ICA, arranged by brain systems. All maps are thresholded at z = 7 (p < 0.00001). (a) Sensorimotor cortex. (b) Polymodal association cortex. (c) Thalamus and hypothalamus. (d) Amygdala. (e) Striatum. (f) Hippocampus. (g) Midbrain.

In this section, we demonstrate functional networks revealed by a data-driven method (ICA). Using the GIFT toolbox, we ran spatial group ICA on all preprocessed rsfMRI scans with the number of components set at 30. Two components were pertaining to the WM and discarded. Fig. 6 shows the spatial patterns of all ICA components, thresholded at z > 7 (p < 0.00001).

**Fig. 7.**
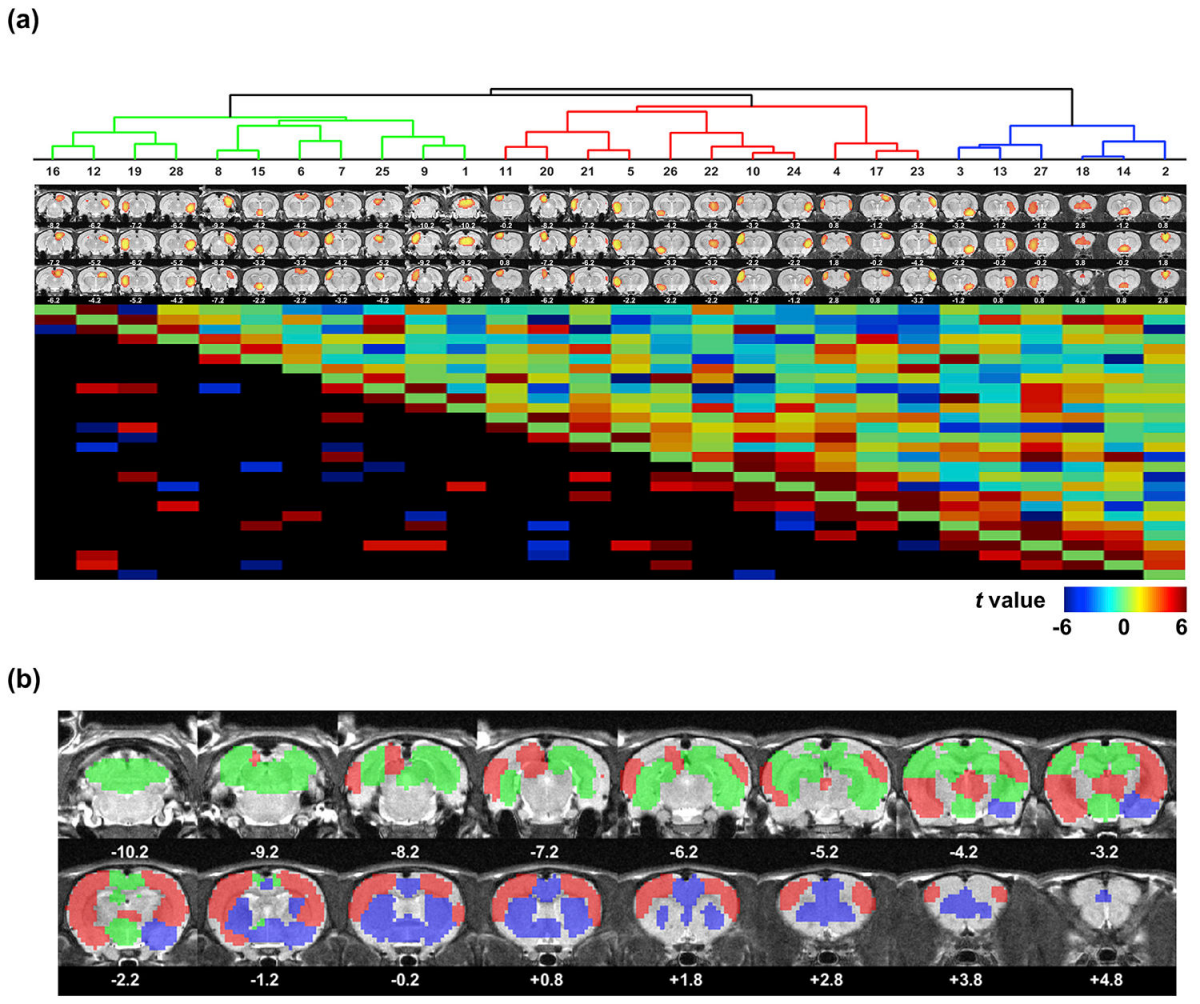
Connectional structure between all ICA components. (a) Hierarchical clustering of ICs (upper panel), and between-IC FC matrix (lower panel). The dendrogram was cut off with an empirical threshold, resulting into 3 modules. The lower triangle shows connections with significant FC thresholded at FWER <0.05. (b) Community structures revealed by color coded ICs based on their corresponding communities (z > 7).

We also demonstrate the connectivity architecture between ICA components. FC between every two ICA components was determined by the Pearson correlation between their time courses for each rsfMRI scan. Group-level inference was determined using the same linear mixed model, resulting in a *t* value for each pair of ICA components, displayed in Fig. 6a. The lower triangle of the *t* matrix shows significant entries (i.e. between-component connections) thresholded at FWER <0.05 with the same permutation test. Subsequently, all components were hierarchically clustered with the Ward’s method (Murtagh and Legendre, 2014) using the FC as the similarity. The dendrogram is shown in Fig. 7a (top), where we cut off the dendrogram with an empirical threshold, resulting in 3 modules (Fig. 7b). The red module mainly consists of the sensorimotor cortex and thalamus, the blue module mainly consists of the frontal-limbic system including the orbital cortex (ORB), prelimbic cortex (PL), infralimbic cortex (IL), ACA, and striatum, and the green module mainly consists of the RSP, hippocampus, hypothalamus, VIS, and dorsal midbrain and hindbrain.

### 3.4. Effects of nuisance signal regression

**Fig. 8.**
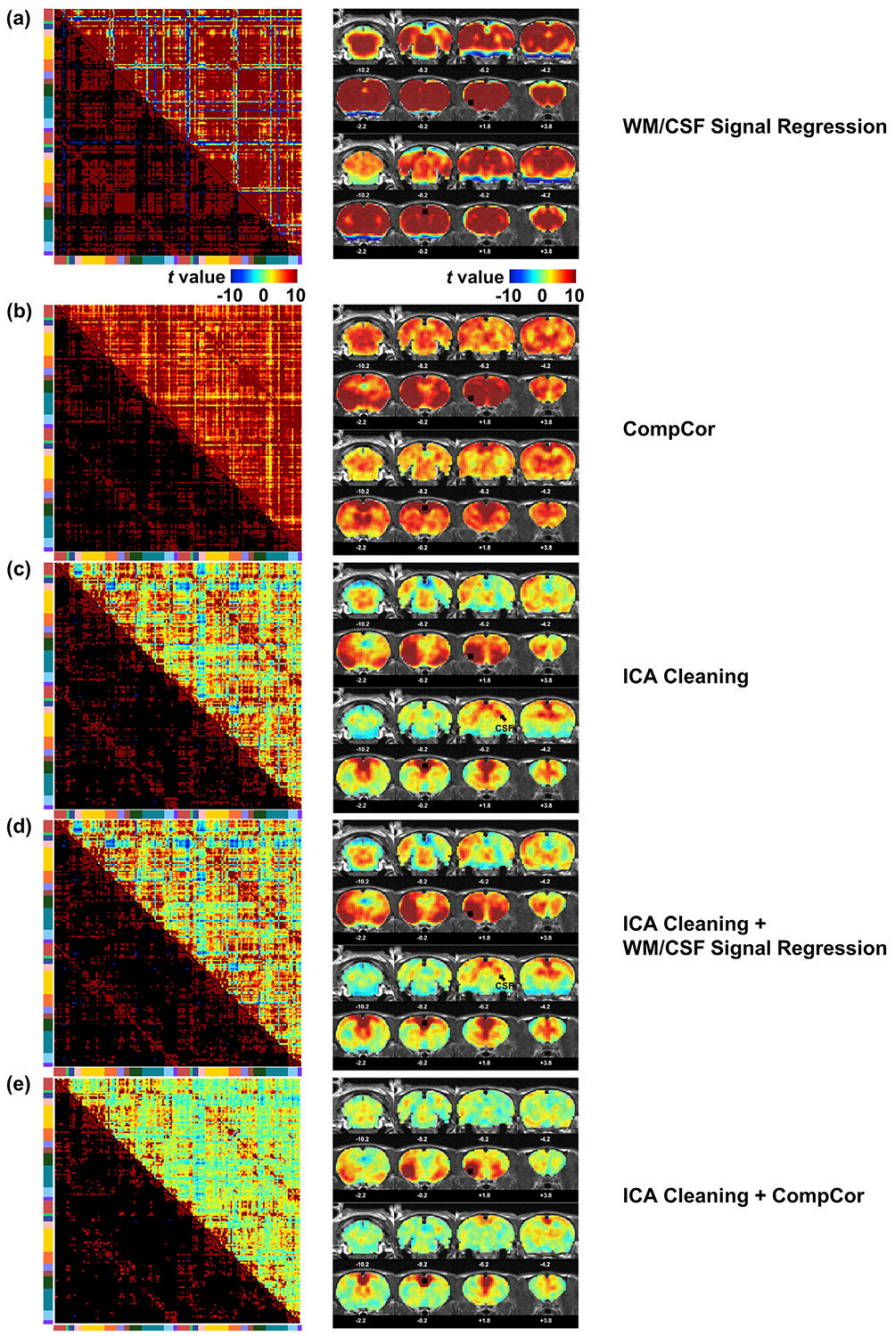
Comparison among different nuisance regression methods. For each subfigure, the left column shows the FC matrix with the same ROI definition as in Fig. 4; The right column shows two seedmaps of the insula (upper) and ACA (lower). (a) WM/CSF signal regression. (b) CompCor. (c) ICA cleaning. (d) ICA cleaning with WM/CSF signal regression. (e) ICA cleaning with CompCor.

Finally, we examined the performance of different nuisance signal regression methods. All motion parameters were regressed out first before testing. We compared the effects of applying WM/CSF signal regression, CompCor, ICA cleaning, ICA cleaning with WM/CSF signal regression, and ICA cleaning with CompCor. Fig. 8 shows the FC matrix with the same ROI definition as in Fig. 4 (left column), and two seedmaps (right column) of the insula (upper) and ACA (lower). These data demonstrate that the preprocessing pipeline we used (i.e. ICA cleaning with CompCor) was able to remove artefacts, but also reveal specific FC in seedmaps.

## 4. Discussion

Neuroscience and psychiatric research have been substantially facilitated by open neuroimaging datasets (Poldrack and Gorgolewski, 2017; Thompson et al., 2014; Van Essen et al., 2013). Data sharing not only speeds up scientific discoveries by leveraging a high statistical power brought by large volumes of data, but also incentivizes researchers to develop new analysis methods that can be tested on these datasets. While a large number of open databases of human rsfMRI studies have been established, such database in rodents, particularly awake rodents is rare (Grandjean et al., 2020). Considering that rodent models are an important translational tool for clinical and basic neuroscience research, here we share an open rsfMRI database acquired in 90 awake rats, and describe the data acquisition protocol and preprocessing procedures.

There has been growing interest in studying brain function and organization in awake rodents using rsfMRI, which avoids interference of anesthesia and permits correlation to behavioral data (Bergmann et al., 2016; Brydges et al., 2013; Chang et al., 2016; Liang et al., 2011; Stenroos et al., 2018; Yoshida et al., 2016). One major challenge of awake rodent fMRI is to control motion and stress during data acquisition. We addressed the issue in three aspects: first, we used a 3D-printed head restraint system to limit animals’ head motion; second, we adopted a 7-day acclimation routine prior to imaging, which has been shown to significantly reduce stress during image acquisition (King et al., 2005); third, we used stringent data preprocessing including scrubbing volumes with excessive motion, regressing out motion parameters, WM/CSF signals, and non-neural artefacts using ICA cleaning. The preprocessing toolbox developed for this database has been open sourced and made publicly available. Our data demonstrate high inter-subject reproducibility in the whole-brain FC matrix both at the group level and the individual level. We showcase inter-regional FC and functional networks calculated from the database. In our library we included seed maps from all individual anatomical ROIs. Taken together, the database shared should provide a resource for comprehensively studying circuit- and network-level function and organization in the awake rodent brain. Such information will not only help us understand the rat brain function, but also be valuable for studies of comparative neuroanatomy. In addition, when linking to information obtained using other tools such as tract tracing, gene expression association, and diffusion tensor imaging, our dataset will open a new avenue to investigate the function-anatomy relationship and perhaps the genetic basis of rsfMRI data. Furthermore, the dataset can potentially provide information to guide the design of studies involving electrophysiology, optogenetics, and behavioral tests in rodents. As more datasets of rodent rsfMRI, potentially collected in different animal models of brain disorders or under different physiological conditions (e.g. anesthesia), become available, these data can be integrated for further investigations of circuit- and network-level changes in these models.

There are a couple of notable limitations in this dataset. First, physiological signals such as respiration and heartbeat were lacking. These signals are useful for removing non-neural noise in the rsfMRI signal, and could also reflect the status of the animal. Second, even though the acclimation procedure has been shown to facilitate the adaptation to the restrainer, the stress response during imaging is always a factor that needs to be considered. It also needs to be recognized that imaging animals in the anesthetized state remains to be a very important method. The choice between these different imaging methods should solely depend on the scientific question asked.

## Supplementary Material

1

## Figures and Tables

**Fig. 9.**
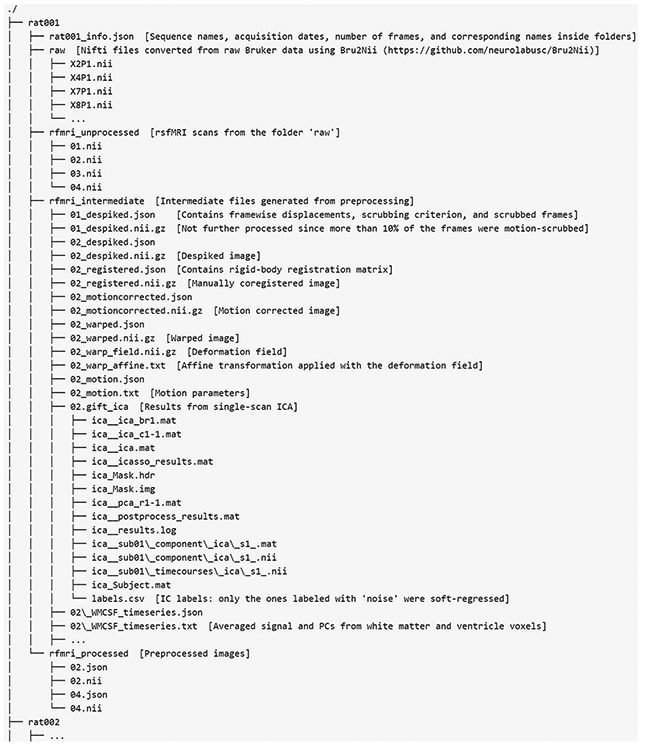
Folder structure of the database.

**Table 1:** Features of signal- and noise-related independent components.

	Human standards		Adaptation for rats
Features	Signal	Noise	Noise
**Spatial features Number and dimension of clusters**	Low number of large clusters	Large number of small clusters	Not applicable
**Overlap with GM**	Clusters’ peaks in GM and overall good overlap of the clusters with GM.	Indiscriminate overlap with non-GM tissues, or clusters’ peaks in WM/CSF	Applicable
**Overlap with WM, CSF, blood vessels**	Very low or on overlap with WM, CSF, blood vessels	High overlap with WM, CSF and/or blood vessels	Applicable
**Overlap with brain boundaries or areas close to the edges of the FOV**	Very low or no overlap with brain boundaries. Clusters follow known anatomical (e.g. structural/ histological) boundaries.	Ring-like or crescent shape or stripes near the edges of the field-of-view	Applicable
**Location near area of susceptibility induced signal loss**	Generally located away from these areas	Located within the region of signal loss (e.g. areas of air-tissue interface)	Confident
**Non-biological, acquisition-related patterns**	Patterns have no relation to acquisition parameters	Often show banding patterns in slice direction or streaks along the phase encoding direction, accelerated sequences may have centrally located artefacts	Confident
**Temporal (and spectral) features**			
**Overall aspect of the time series**	Fairly regular/oscillatory time course	Large jumps and/or sudden change of oscillation pattern.	Applicable
**Distribution of power in frequency domain**	Predominantly low frequency (at least one strong peak within 0.01–0.1 Hz)	Predominantly high frequency, very low frequency, or pan frequency	Applicable

## Appendix

### Folder structure of the database

Fig. 9 shows the folder structure of the database. Data of each subject are placed in a single folder named ‘rat’ followed by the subject index ‘xxx’. In each folder, raw data, unprocessed rsfMRI scans, preprocessed scans, and intermediate files from preprocessing are separately placed in the folders named ‘raw’, ‘rfmri_unprocessed’, ‘rfmri_processed’, and ‘rfmri_intermediate’, respectively. All image files are in the NIfTI (Neuroimaging Informatics Technology Initiative) format. Also, the sequence name and acquisition time of each scan, as well as their names in each folder are stored in the file named ‘ratxxx_info.json’ in the JSON (Java-Script Object Notation) format. In the ‘rfmri_intermediate’ folder, despiked, rigid-body registered, motion-corrected, and images obtained using deformable registration (warped) are provided, as well as framewise displacement values and scrubbed frame indices from the despiking step (in the files ended with ‘_despiked.json’), transformation matrix for the rigid-body registration (in the files ended with ‘_registered.json’), motion parameters (in the files ended with ‘_motion.txt’), deformation field for the deformable registration (in the files ended with ‘_warp_field.nii.gz’ and ‘_warp_affine.txt’), the average and principal components (selected by the CompCor method) of WM/CSF signals (in the files ended with ‘_WMCSF_timeseries.txt’) from warped images. Results from single-scan ICA cleaning and IC labels are placed in the folder ended with ‘.gift_ica’. Each image file in the ‘rfmri_intermediate’ and ‘rfmri_processed’ folders is accompanied with a JSON file containing the processing steps completed and parameters used.
