# Supplementary material for: An open database of resting-state fMRI in awake rats
Source: Neuroimage. Author manuscript; Available in PMC 2020 Nov 2. (PMC7605641; doi:10.1016/j.neuroimage.2020.117094)
Supplement: 1 [file NIHMS1639062-supplement-1.pdf]

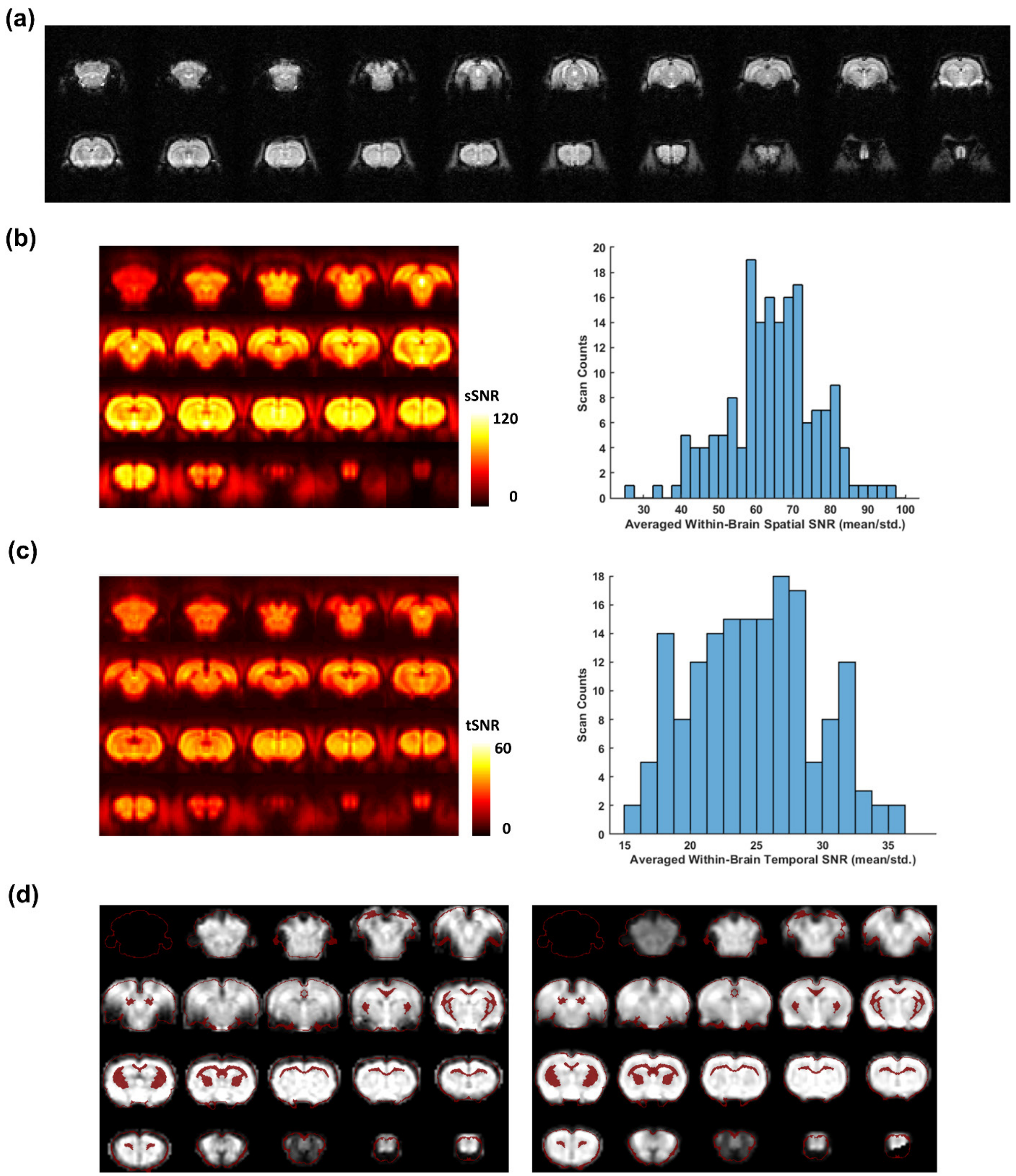

Figure S1. Data and deformable registration quality. (a) A representative raw EPI frame. (b-c) Spatial and temporal SNR. The left panels show the sSNR and tSNR maps averaged across scans. The right panels show the distributions of within-brain averaged SNR across scans. (d) A representative example of deformable registration. The left panel shows an EPI frame before deformable registration; the right panel shows the same frame after deformable registration.

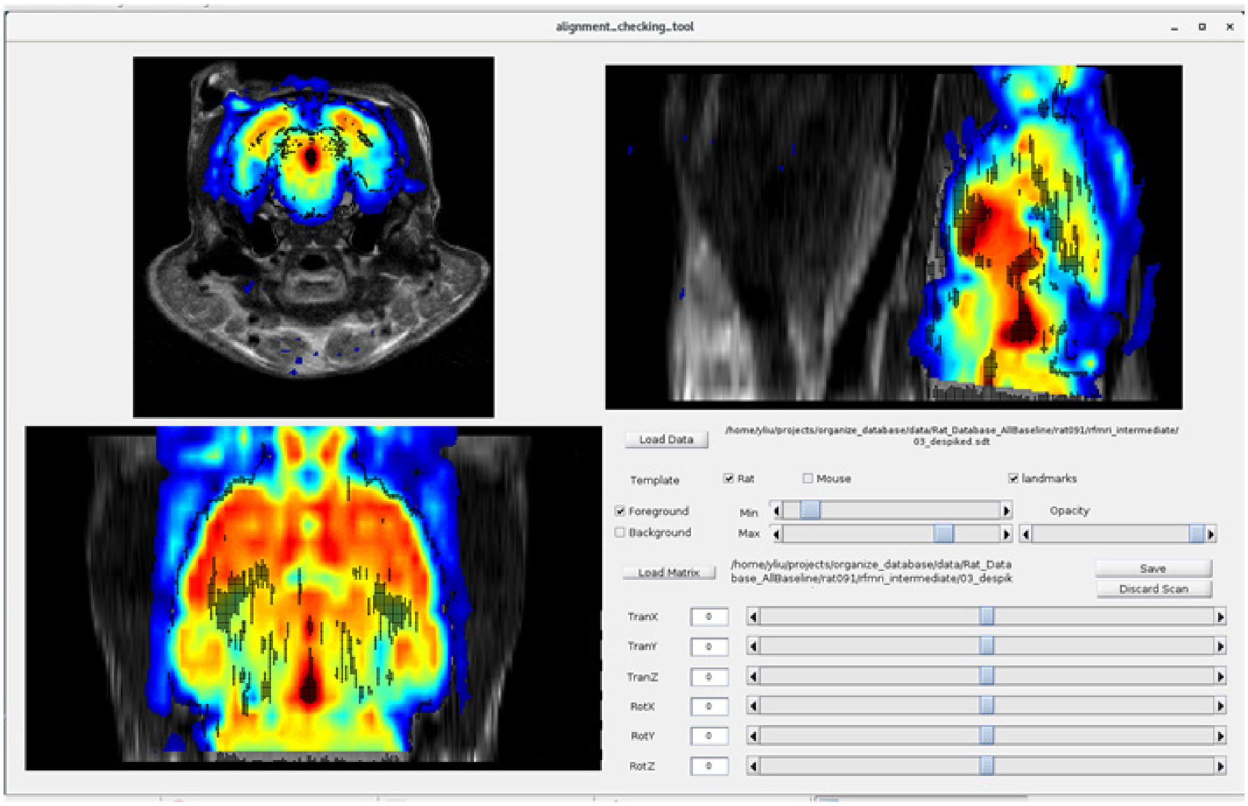

Figure S2. User interface of the linear coregistration toolbox.

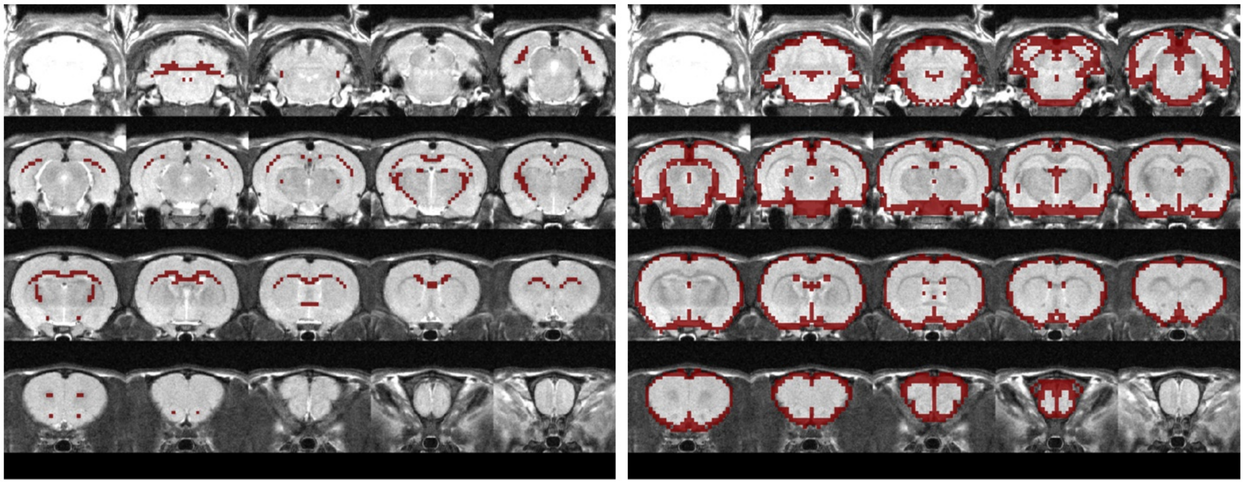

Figure S3. White matter (left) and CSF (right) masks.

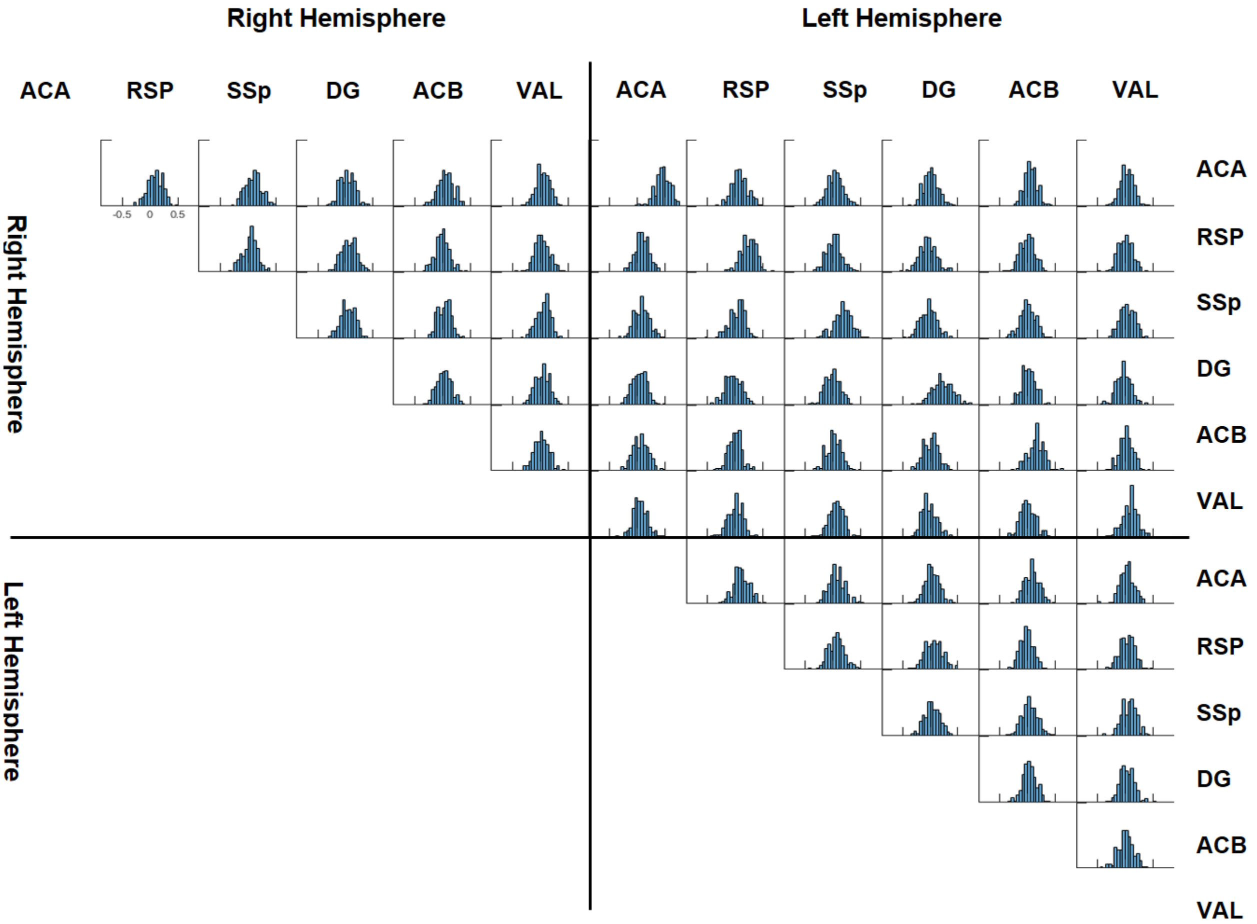

Figure S4. Distributions of FC between selected ROI pairs.

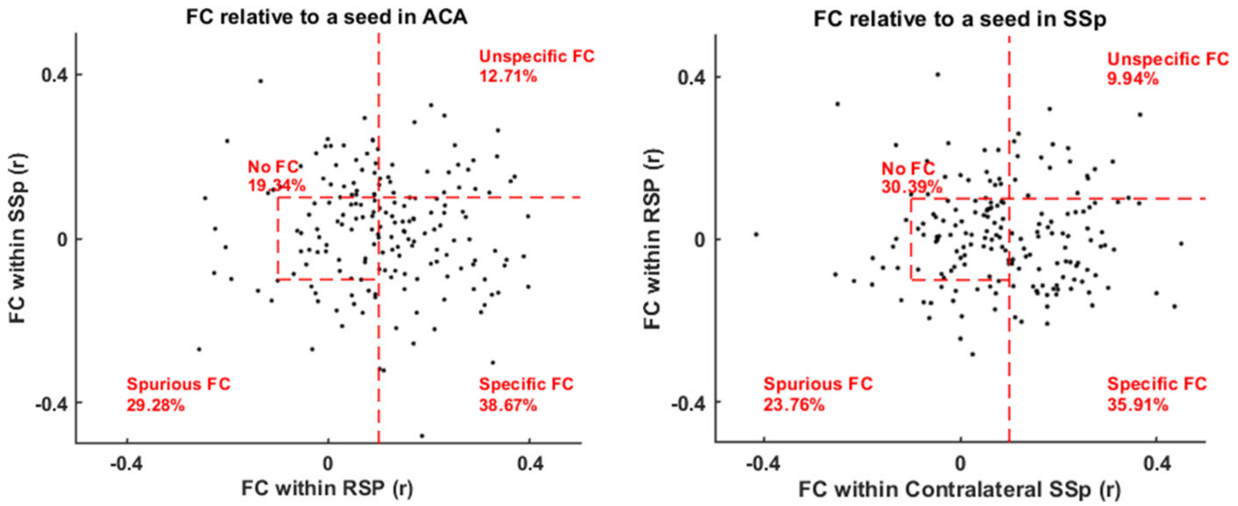

Figure S5. FC specificity. In the left panel, ACA, RSP, and SSp are the seed, specific ROI, and non-specific ROI, respectively. In the right panel, SSp, contralateral SSp, and RSP are the seed, specific ROI, and non-specific ROI, respectively. Each seed region is expected to have positive FC with the specific ROI, but low or negative FC with the non-specific ROI. A scan shows "specific FC" if the seed has positive FC with the specific ROI and low or negative FC with the non-specific ROI; shows "unspecific FC" if the seed has positive FC with both specific and non-specific ROIs; shows "spurious FC" if the seed has low or negative FC with the specific ROI and positive or negative FC with the non-specific ROI; and shows "no FC" if the seed has low FC with both ROIs.
